# *Saprolegnia diclina IIIA* and *S. parasitica* employ different infection strategies when colonizing eggs of Atlantic salmon, *Salmo salar* L.

**DOI:** 10.1111/jfd.12368

**Published:** 2015-04-07

**Authors:** M M Songe, A Willems, J Wiik‐Nielsen, E Thoen, Ø Evensen, P van West, I Skaar

**Affiliations:** ^1^ Norwegian Veterinary InstituteOsloNorway; ^2^ Aberdeen Oomycete LaboratoryCollege of Life Sciencesand Medicine, Institute of Medical SciencesUniversity of AberdeenAberdeenUK; ^3^ Faculty of Veterinary Medicine and BiosciencesNorwegian University of Life SciencesOsloNorway

**Keywords:** chorion disruption, egg infection, infection strategies, *Saprolegnia*

## Abstract

Here, we address the morphological changes of eyed eggs of Atlantic salmon, *Salmo salar* L. infected with *Saprolegnia* from a commercial hatchery and after experimental infection. Eyed eggs infected with *Saprolegnia* spp. from 10 Atlantic salmon females were obtained. Egg pathology was investigated by light and scanning electron microscopy. Eggs from six of ten females were infected with *S. parasitica*, and two females had infections with *S. diclina* clade IIIA; two *Saprolegnia* isolates remained unidentified. Light microscopy showed *S. diclina* infection resulted in the chorion in some areas being completely destroyed, whereas eggs infected with *S. parasitica* had an apparently intact chorion with hyphae growing within or beneath the chorion. The same contrasting pathology was found in experimentally infected eggs. Scanning electron microscopy revealed that *S*. *parasitica* grew on the egg surface and hyphae were found penetrating the chorion of the egg, and re‐emerging on the surface away from the infection site. The two Saprolegnia species employ different infection strategies when colonizing salmon eggs. *Saprolegnia diclina* infection results in chorion destruction, while *S. parasitica* penetrates intact chorion. We discuss the possibility these infection mechanisms representing a necrotrophic (*S. diclina*) vs. a facultative biotrophic strategy (*S. parasitica*).

## Introduction

Aquaculture has become the world's fastest growing food sector. With increasing production intensity, the control and reduction of health problems in the entire production chain is paramount for future success of the aquaculture industry. It has been established that the greatest losses of fish eggs are caused by infection with *Saprolegnia* species (Willoughby 1970; Czeczuga & Kiziewicz 1999; Hussein, Hatai & Nomura 2001). The use of malachite green, a very effective treatment against *Saprolegnia* infections (Fitzpatrick *et al*. 1995; Kitancharoen, Yamamoto & Hatai 1997), was banned worldwide due to its carcinogenic and toxicological effects. At present, there is no equally efficient treatment available, and therefore, saprolegniasis has become an increasing problem worldwide. It has been estimated that over 10% of salmonid eggs become infected with oomycetes in hatcheries each year (Bruno, Van West & Beakes 2011). In Norwegian salmon farming, *Saprolegnia* infection is mainly a problem in incubating eggs and newly hatched fry (Thoen, Evensen & Skaar 2011). Outbreaks are also seen in fingerlings and parr throughout the freshwater stage, although more sporadically. In other countries with large‐scale salmonid farming such as Chile, Japan and Scotland, the problem is also present during the egg‐incubation stage (Beakes, Wood & Burr 1994; Kitancharoen & Hatai 1996, 1997; Kitancharoen *et al*. 1997; Hussein *et al*. 2001; Van Den Berg *et al*. 2013). Numerous studies of *Saprolegnia* infection in immature and mature stages of salmonids have been conducted (Wood, Willoughby & Beakes 1988; Pottinger & Day 1999; Hussein & Hatai 2002; Stueland, Hatai & Skaar 2005; Thoen *et al*. 2011). However, only a few scientific reports of pathogenesis of *Saprolegnia* infections in salmonid eggs are available (Kitancharoen & Hatai 1996; Thoen *et al*. 2011). In particular, the role of *S. parasitica* as a cause of saprolegniasis in fish eggs is still unclear. Fish eggs are thought to be killed by hypha breaching the chorionic membrane regulating the osmosis of the embryo (Liu *et al*. 2014). A better understanding of the infection process would enable us to develop new sustainable control strategies against infection and can create the basis for the development of new therapeutic interventions. The aim of this study was to determine which species of *Saprolegnia* was the most prevalent cause of egg infection under natural conditions, and to better understand whether the two *Saprolegnia* species employ different infection strategies under these circumstances and following an experimental infection of eggs.

## Materials and methods

### Atlantic salmon eggs

Living eyed eggs, 280 degree‐days old and visibly infected with *Saprolegnia* originating from 10 different Atlantic salmon, *Salmo salar* L. females were collected from Landcatch, a commercial hatchery located in Scotland. The different females from which the eggs were collected are treated as biological replicates and hence are kept in different units in the hatchery. The unit numbers were noted and recorded for possibility of lineage tracing. The eggs were considered infected if they were tightly clumped together in a tuft of mycelium. From each female, seven infected eggs were collected from each female (*n* = 70). One egg from each female (*n* = 10) was immediately placed on glucose–yeast extract (GY) agar consisting of 1% glucose, 0.25% yeast extract and 1.5% agar (Hatai & Egusa 1979) for cultivation, isolation and identification of the *Saprolegnia* isolates involved. Three of the 6 remaining eggs (*n* = 60) were fixed in 15 mL of 10% phosphate‐buffered formalin (Bancroft & Stevens 1990) and kept at 4 ± 1 °C. The last three eggs (*n* = 30) were fixed in 15 mL of 4% paraformaldehyde/1% glutaraldehyde/phosphate buffer and kept at 4 ± 1 °C (Glauert & Lewis 1998). The fixatives were replaced with freshly prepared solutions of 10% phosphate‐buffered formalin and 4% paraformaldehyde/1% glutaraldehyde/phosphate buffer after 24 h and stored until further processing. Additionally, one uninfected egg was collected from each female to serve as the negative control for the histology.

### Purification of the *Saprolegnia* isolates

An agar plug of 5 mm in diameter, colonized by *Saprolegnia* hyphae, was cut from the GY plate and transferred to another plate containing GY agar (Hussein & Hatai 1999). To inhibit bacterial growth, the GY agar was supplemented with 200 μg mL^−1^ chloramphenicol (Fregeneda‐Grandes, Rodríguez‐Cadenas & Aller‐Gancedo 2007). The growing hyphae were cut into small pieces and transferred to sterile aquarium water (SAW) for zoospore production. Single‐spore isolations were performed on GY agar with chloramphenicol and incubated at 21 ± 1 °C for 2–5 days (Onions, Allsopp & Eggins 1981). These procedures were repeated until pure cultures were obtained. Pure cultures were stored on autoclaved hemp seeds at 4 ± 1 °C according to the procedure described earlier (Stueland *et al*. 2005).

### Morphological identification

The purified strains were identified morphologically (Willoughby 1970, 1986). From a single‐spore culture on GY agar, a 5‐mm‐diameter plug of the growing mycelium was placed in GY broth (Hatai & Egusa 1979; Kitancharoen *et al*. 1995) and incubated for 2–3 days at 15 ± 1 °C. Bundles of hyphae were washed with SAW and incubated with autoclaved hemp seeds in SAW. Examination of possible sexual structures on hemp seeds was performed at 5, 15 and 20 ± 1 °C, and the hemp seed cultures were examined for production of oogonia and antheridia twice a week over a 12‐week period using an Olympus^®^ inverted zoom stereo microscope (SZH‐ILLD), with a bright field/dark field transmission light illumination base (Stueland *et al*. 2005).

### Molecular identification

The purified *Saprolegnia* isolates from infected Atlantic salmon eggs were subjected to molecular identification. Genomic DNA was extracted from 20 mg mycelia from each isolate using CTAB miniprep extraction protocol (Gardes & Bruns 1993). The ITS region was amplified using the universal fungal reverse primer ITS4 (White *et al*. 1990) and oomycete specific forward primer ITS1_Omyc. The 25 μL reaction mixture consisted of 1.7 μm of each primer, 2 μL of genomic DNA, puReTaq Ready‐To‐Go PCR Beads (Amersham Biosciences) and milliQ water. PCR was performed on a Bio‐Rad (Biorad/MJR) DNA Engine Dyad thermal cycler. The PCR amplicons were visualized by gel electrophoresis on 1.0% agarose gel stained with Gelred (Huang *et al*. 2010). PCR products were purified with ExoSAP‐IT (Amersham Bioscience) according to the protocol. The products were then sequenced in both directions with their respective primers, using the BigDye^®^ Terminator v3.1 Ready Reaction Mix (Applied Biosystems, Life Technologies). The sequenced PCR products were purified with BigDye^®^ XTerminator Purification kit (Applied Biosystems, Life Technologies) according to manufacturer's instructions and subsequently analysed on an ABI PRISM^®^ 3100 – Avant Genetic Analyzer (Applied Biosystems). Sequence contigs were assembled and quality‐controlled in BioEdit (Hall 1999). The sequences were compared to publically available sequences using the NCBI nucleotide Basic Local Alignment Search Tool (BLAST) (Altschul *et al*. 1997) and identified on the basis 100% identity to well‐annotated *Saprolegnia* reference strains.

### Sample processing

For light microscopic examination, the infected and negative control eggs, fixed in 10% phosphate‐buffered formalin, were treated as follows: dehydration through ascending alcohol grades, clearing in xylene, impregnation with wax, cutting at 5 μm, mounting on a glass slide, complete de‐waxing and staining with haematoxylin and eosin. The microscopic slides were the examined with a light microscope.

The disruption of the chorion was the main finding, and the changes seen were categorized (subjectively) into three different grades: minor, moderate and severe. Minor changes were observed as single foci damage to the chorion with the loss of continuity of the chorion surface without these changes extending all the way through the chorion. These were found with hyphae on the surface of penetrating into the chorion. Moderate changes included loss of continuity over a larger area and typically with pore formations in the chorion. Hyphae were found on the surface and penetrating into the chorion. Severe changes were complete loss of continuity, disorganized chorion and exposure to underlying structures. Hyphae were located on the surface and growing into the layers beneath the chorion. Formation of cysts was also recorded. The degree of vacuolization of the egg cytoplasm was also included in the evaluation and was separated into minor (few vacuoles), moderate (increasing number and coalescing) and severe including vacuolation of large areas of the egg cytoplasm.

Scanning electron microscopy. The eggs were fixed in 4% paraformaldehyde/1% glutaraldehyde/phosphate buffer. Post‐fixation was carried out with 1% buffered osmium tetroxide. Subsequent treatments included (i) dehydration in ascending grades of acetone and were dried in tetramethyl silane (TMS) following the method of Dey *et al*. (1989). The dried samples were mounted on brass stub (10 × 30 mm) using a double‐sticky adhesive tape, connected via a patch of silver paint to ensure charge conduction. A thin conductive coating of gold was applied to the samples using JFC 1100 (Jeol) ion sputter at a relatively low vacuum of 10^−3^ torr, or (ii) air‐drying and direct conductive gold coating as described. The coated samples were examined in a Zeiss EVO 50 EP Scanning Electron Microscope at an accelerating voltage of 20 kV in the secondary electron emission mode.

### Salmon eggs for experimental infection

Eyed eggs from Atlantic salmon, *Salmo salar* L. of strain AquaGen Atlantic QTL‐innOva IPN from AquaGen AS were used for the experimental infection. The eggs were 385 degree‐days on the day of shipment. They were disinfected during incubation and before transport with buffodine (1:100, 10 min) and treated with formalin according to AquaGen's in‐house protocols. The eggs were gradually (approximately 24 h) brought to the laboratory water temperature used during the experiments, which varied from 8.7 to 9.6 °C (for the live egg experiments; described below). The eggs were then acclimatized for 3 days in the laboratory to avoid mortality from transportation damage, prior to the challenge experiments. Blank, pin‐eyed and white eggs were removed if observed.

### *Saprolegnia* strains used for experimental infections

Two strains of *Saprolegnia* spp (*S. parasitica* VIO 2741 and *S. diclina* clade IIIA, VIO 2739) previously shown to be pathogenic to Atlantic salmon eggs (Thoen *et al*. 2011) were used for the artificial infections. Cysts were produced according to the method described by Stueland *et al*. (2005). The cysts were counted using a haemocytometer (Bürker turk) and the cyst suspensions adjusted by dilution to obtain the required density 1 × 10^4^ spores L^−1^ (Thoen *et al*. 2011).

### Preparation of infected dead eggs (focus of infection)

Adopting methods used by Thoen and coworkers (Thoen *et al*. 2011), groups of live, eyed eggs were killed by immersion for 1 min in water bath at temperature of 60 °C. The dead eggs were incubated in *Saprolegnia* spore suspensions (1.0 × 10^4^ spores L^−1^) in 24‐well microwell plates at 15 °C for 48 h. Incubated eggs were examined microscopically for the presence of *Saprolegnia* hyphae.

### Challenge of live eggs by co‐incubation with pre‐infected dead eggs

Live eggs were assigned to duplicate groups of 100 ± 2 eggs each per *Saprolegnia* isolate and distributed in two separate compartments in small‐scale hatching trays (one tray with two compartments of 100 eggs for each of the *Saprolegnia* species) with a flow‐through system. The eggs were spread to form an even layer covering the bottom of the trays. In each compartment, groups of four eggs pre‐infected with the respective isolates, as previously described, were placed on the layer of live eggs.

The four infected eggs per compartment were carefully placed in the corners of an imagined square on the layer of live eggs. This was carried out to let the hyphae from each of the infected dead eggs having the opportunity to infect an approximately equal number of the 100 live eggs. In addition to the groups with infected eggs, two groups of four dead eggs that were not exposed to *Saprolegnia* spores were introduced in compartments with 100 live eggs to serve as non‐infected control groups. The experimental units were maintained and inspected daily for 10 days.

At termination of the experiment, the number of live eggs newly infected by hyphae from each of the introduced dead eggs was counted for each isolate. The live eggs were considered as infected and counted when they were entangled in hyphae and did not unfasten when the dead eggs were moved. At termination of the experiment (day 10 post‐infection), representative samples of infected eggs were fixed in 10% phosphate‐buffered formalin, embedded in paraffin and processed for examination by light microscopy, as described previously for the naturally infected eggs.

## Results

### Identification of *Saprolegnia* strains

The isolates infecting the collected eggs were identified by means of molecular and morphological methods. Two *S. diclina* and six *S. parasitica* isolates were collected and grown in pure culture from eggs from eight different fish (Table 1). Two of the *Saprolegnia* isolates could not be successfully purified due to recalcitrant contaminations with yeasts and bacteria.

**Table 1:** Molecular identification of *Saprolegnia* isolates

Female ID	*Saprolegnia* species	NVI culture collection number	GenBank accession number
1	*S. diclina clade IIIA*	VI0 6011	HG329742
2	*S. diclina clade IIIA*	VI0 6008	HG329735
3	*S. parasitica*	VI0 5977	HG329736
4	*S. parasitica*	VI0 5978	HG329737
5	*S. parasitica*	VI0 6009	HG329739
6	*S. parasitica*	VI0 5979	HG329738
7	*S. parasitica*	VI0 5980	HG329740
8	*S. parasitica*	VI0 5981	HG329741
9	Unidentified	–	–
10	Unidentified	–	–

### Histopathological changes in field‐collected eggs

**Figure 1:**
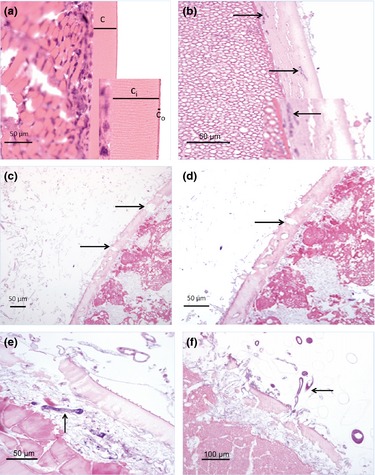
Histology of normal (a) and histopathology of infected eggs (b–f). (a) Healthy chorion separated into an outer thin layer (c_o_; insert) and inner thicker layer (c_i_). The cell‐rich layer inside of the chorion is likely part of the blastoderm. (b) Hyphae of *S. parasitica* are located inside the chorion, which shows minor changes. The hyphae are located in the mid‐part and towards the yolk granules (arrows). Details of the hyphae are shown in the insert (arrow). (c) *S. parasitica* infection with moderate chorion changes. Numerous hyphae on the outside of the egg, and there are several pores and vacuoles seen in the chorion wall (arrows). (d) Higher magnification of (c) detailing the vacuoles and the cracks in the chorion (arrow). Note numerous hyphae. (e) *S. diclina* infection, with moderate‐to‐severe changes of the chorion (and cytoplasm). Germinated cysts present below the cracked chorion and inside the egg (arrow). (f) *S. diclina* infection, severe chorion changes. Almost a complete wipeout of the chorion in some areas and with thinner chorion than normal in others. Chorion is also discontinuous and changes are associated with the presence of hyphae (arrow). Bars = 50 μm.

Examination by light microscopy of eggs from the non‐infected control group showed 2 intact layers of the chorion. The outer envelope is very thin and was stained dark pink, while the inner layer was pale pink and with thin radial lines (Fig. 1a). The cytoplasm was composed of yolk granules, and areas with blastomeres were also seen. The surface of the normal eggs was smooth and without any loss of integrity at the perimeter (Fig. 1a).

Examination of infected eggs showed clear differences between *S. parasitica‐* and *S. diclina‐*infected eggs. Eggs infected with *S. parasitica* varied and had an intact or minor‐disrupted chorion, that is minor changes (Fig. 1b). Despite the chorion being intact, *Saprolegnia* hyphae could be observed inside the eggs from four females 6–9 (Fig. 1b, Table 2). Some eggs were found with numerous pores and cracks in their chorion (categorized as moderate changes) and with hyphae that had penetrated through the chorion and accumulated in the cytoplasm, where vacuolization was evident, that is moderate changes (Table 2, Fig. 1c).

**Table 2:** Microscopic findings in infected ova of Atlantic salmon, *Salmo salar* L

Female number	*Saprolegnia* species involved	Grade of disruption of chorion	Light microscopy findings				Scanning electron microscopy (SEM) findings	
			Localization of hyphae		Vacuolation of cytoplasm	Germinated cysts present inside the egg	Localization of hyphae and/or germinated cysts	
			Egg surface	Inside			Egg surface	Penetration into chorion
1	*S. diclina*	Severe	+++	+++		++	Observed	Observed
2	*S. diclina*	Severe	+++	+++	Severe	++	No SEM micrograph	‐
3	*S. parasitica*	Mild	++	++	Mild	+	Observed	Observed
4	*S. parasitica*	Moderate	+++	+++	Not observed	+	Observed	Not observed
5	*S. parasitica*	Mild	+++	+	Not observed	+	Not observed	Not observed
6	*S. parasitica* a	No	++	++	Not observed	Not observed	Observed	Not observed
7	*S. parasitica* a	No	++	++	Not observed	+	Observed	Not observed
8	*S. parasitica* a	No	++	++	Not observed	+	Observed	Observed
9	NI^a^	No	++	++	Moderate	+	Observed	Observed
10	NI	Mild	++	++	Not observed	Not observed	Observed	Observed

NI, not identified.

a Hyphae inside egg with intact chorion on histopathology.

Eggs of females (1 and 2) infected with *S. diclina* displayed severe changes with a chorion that was almost completely destroyed and in parts indiscernible (Fig. 1e,f). Germinated cysts were observed inside the egg, that is beneath the chorion or what was left of the chorion (Fig. 1e, Table 2) and with hyphae radiating out from the chorion (Fig. 1f). The chorion was discontinuous, only partly intact (Fig. 1f).

**Figure 2:**
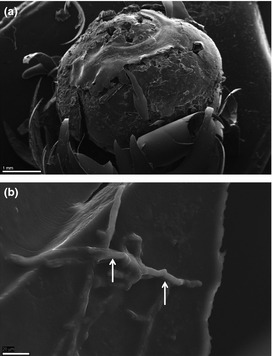
SEM of an infected egg. (a) Egg cracked open during processing, separating the outer and inner layer, thus exposing the inner surface. Bar = 1 mm. (b) *Saprolegnia parasitica* hyphae invading the inner surface of the outer layer of the chorion (arrows). Bar = 20 μm.

**Figure 3:**
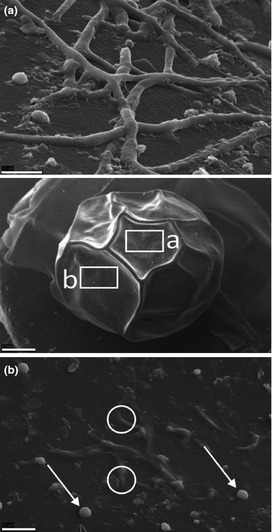
SEM of an air‐dried infected egg (in the middle) Bar = 1 mm. A close‐up of the areas marked a (upper) and b (lower) shows *Saprolegnia parasitica* cysts (arrows) and hyphae growing on the outer surface of the egg. Cysts (arrows) are also visible. Hyphae are seen penetrating into the chorion (circles). Bars a and b = 20 μm.

By scanning electron microscopy due to the eggs cracking during preparation (Fig. 2a), it was possible to show *Saprolegnia* hyphae on the inner side of the outer envelope (Fig. 2b). We also employed air‐drying of the eggs, and on intact eggs, *Saprolegnia* hyphae were observed on the surface of the egg (Fig. 3b). Further, hyphae also seemed to penetrate into the chorion of the egg and re‐emerge on the surface (Fig. 3c). No appressoria‐like structures were observed.

### Experimental infection results

The high prevalence of *S. parasitica* infection in eyed eggs was a surprising finding, as was the contrast in pathology and infection dynamics observed between the two species under field conditions. For this reason and to ascertain that environmental factors in the hatchery had not selected for *S. parasitica* strains with particular virulence profiles, an infection experiment was carried out in the laboratory and included *S. parasitica* and *S. diclina* clade IIIA. The origin of these strains was different from what was observed in the field experiment.

Examination of the histopathological changes from experimentally infected eggs showed differences in egg pathology between *S. parasitica* and *S. diclina* that corroborated the findings from the field experiment. As it was difficult to demonstrate the early stages of infection from the field samples, particularly for *S. diclina*, we focused particularly on this stage for the experimental study.

**Figure 4:**
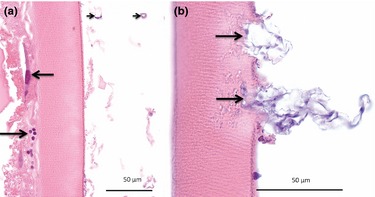
(a) *Saprolegnia parasitica*‐infected egg showing an intact or moderately disrupted chorion and with hyphae located on the outside and/or on the inside of an intact chorion and chorion changes scored as ‘mild’. Remnants of hyphae were seen on the outside of the chorion (arrowheads) without the outer membrane loosing its continuity. (b) *Saprolegnia diclina* infection with the outer chorion membrane disrupted and with disintegration of the inner chorion membrane. Hyphae were found attached to the ‘chorion wounds’ and also extending down into the inner chorion membrane (arrows). The radial orientation of the inner chorion membrane was distorted, and small cracks were seen in the inner membrane (arrow). Bar = 75 μm.

Eggs infected with *S. parasitica* had an intact or moderately disrupted chorion and with hyphae located on the outside and/or on the inside of an intact chorion and chorion changes scored as ‘mild’ (Fig. 4a). Remnants of hyphae were seen on the outside of the chorion without the outer membrane loosing its continuity (Fig. 4a). At the early stage of *S. diclina* infection, the outer chorion membrane was found disrupted and also with disintegration of the inner chorion membrane (Fig. 4b). The radial orientation of the inner chorion membrane was distorted, and small cracks were seen in the inner membrane (Fig. 4b). Hyphae were found attached to the ‘chorion wounds’ and also extending down into the inner chorion membrane (Fig. 4b). More advanced stages appeared as for the field samples and are equivalent to what was shown in Fig. 1b–f.

## Discussion

Two *Saprolegnia* species were isolated from infected eyed eggs in this study, *S. parasitica* and *S. diclina clade IIIA*. *Saprolegnia parasitica* was found more prevalent than *S. diclina* as a source of saprolegniasis in eyed eggs from field samples, in contrast to what has been considered the most frequent cause of egg infection (Kitancharoen *et al*. 1997; Fregeneda‐Grandes *et al*. 2007; Van Den Berg *et al*. 2013). Furthermore, we noticed that *S*. *parasitica* hyphae penetrated the egg chorion without destroying it, while *S. diclina‐*infected eggs had a completely disrupted or destroyed chorion. These findings were replicated following experimental challenge with eyed eggs. This would point towards the two species employ different infection strategies when colonizing and infecting eggs of Atlantic salmon (Van West *et al*. 2010; Wawra *et al*. 2012). We propose this might represent a necrotrophic strategy employed by *S. diclina,* which contrasts a possible facultative biotrophic mechanism by *S. parasitica*. Importantly, the *S. diclina* strain detected in field samples and used for experimental infection was of clade IIIA and, together with clade IIIB, these are the most prevalent variants found in Norwegian salmon hatcheries (unpublished results).

Despite differences in preferred hosts, *S. diclina* and *S. parasitica* are closely related (Dieguez‐Uribeondo *et al*. 2007; Sandoval‐Sierra, Martín & Diéguez‐Uribeondo 2014) species, mostly reported from areas with a temperate climate, such as north‐west Europe, Chile, Japan and Canada, where they have a large impact on salmon farming (Van Den Berg *et al*. 2013). Over the years, researchers have described different mechanisms of pathogenesis displayed by *Saprolegnia* and other oomycetes when they infect salmonids and their eggs. Peduzzi and Bozzozero (Peduzzi & Bizzozero 1977) detected a chymotrypsin‐like enzyme system in culture filtrate and mycelial extracts from water moulds associated with saprolegniasis in fish. They observed that an extracellular proteolytic enzyme produced by the oomycete would favour the deep penetration by invading hyphae into the host tissue. Plant oomycetes are able to breach cuticles of host plants and establish infection rapidly (Soanes, Richards & Talbot 2007). First, the pathogen synthesizes the new cell wall to make infection‐associated structures. Secondly, it breaks down the physical barrier of the plant cell wall by both enzymatic action and/or mechanical pressure, depending on the infection strategy of the pathogen (Mach 2008). As a consonance, the structure of the egg chorion and the thickness of the mucus layer covering it play a role in the occurrence of different mycotic species of fish eggs (Lartseva & Altufiev 1987). Rand and Munden (Rand & Munden 1992) suggested that invasion of living fish eggs by pathogenic strains of *Saprolegnia* species is facilitated by a combination of mechanical pressure and enzymatic activities of their mycelia. They detected enzymes on *S. diclina*‐infested brook char eggs and suggested that the enzymes may have altered the integrity of the chorionic membrane by solubilizing structural polymers and facilitating the penetration of the hyphae through the chorion membrane. Our findings concur with these observations for *S. diclina*‐infected eggs, but the presence of *S*. *parasitica* hyphae beneath intact egg chorion is not easily understood. In concert with what Rand and Munden (Rand & Munden 1992) proposed, mechanical pressure might play a role in hyphae penetration but enzymes released from the hyphae might also facilitate penetration, or a combination of the two is also possible. Importantly, we observed the same phenomena from field infections as for experimental challenge with the two *Saprolegnia* species. While *S. diclina* infection results in distinct egg pathology and with severe necrosis of the chorion, *S. parasitica* obviously employ a different strategy. On this basis, it is tempting to speculate whether *S. diclina* possibly employ a necrotrophic strategy, while *S. parasitica* would represent a biotrophic lineage. This can possibly be used as guidance when searching for underlying biological traits, such as enzyme selections, as seen for certain lineages of necrotrophic fungal pathogens (Sprockett, Piontkivska & Blackwood 2011). *Saprolegnia diclina* might represent a lineage‐specific expansion of a pathogen, particularly virulent for salmon eggs. These findings warrant additional studies into the pathogenesis of fish‐egg saprolegniasis (Lartseva & Altufiev 1987).

Several researchers have reported *S. diclina* as the major species infecting fish eggs (Hussein *et al*. 2001). However, our findings are more in line with what was reported by Shahbazian *et al*. (2010), who found that *S. parasitica* was the most frequently detected species associated with egg infections. They also observed noticeable differences in the fungal and oomycete communities at the two hatcheries that they investigated. They asserted that ecological differences resulting from different hatchery conditions (chemical factors, age of broodstock, density of eggs) may have played a role in the type of fungi and oomycetes that developed on the rainbow trout eggs in their study (Willoughby 1986; Hussein *et al*. 2001). Khosravi and coworkers (Khosravi *et al*. 2012) also isolated a higher percentage of *S. parasitica* from infected eggs (54.3%), compared to 45% *Saprolegnia* spp and 0.7% *Fusarium solani* during their evaluation of antifungal activity of various essential oils for treatment of rainbow trout eggs infected with *Saprolegnia sp*. Therefore, it seems safe to state that *S. parasitica* is also an egg‐pathogenic species and that local environmental factors may influence the prevalence of a particular species, but these remain to be identified.

Although the present study only yielded *S. parasitica* and *S. diclina* from the infected Atlantic salmon eggs from the commercial hatchery, other researchers have shown that other species may also be involved in infecting fish eggs. Rezinciuc and coworkers (Rezinciuc, Sandoval‐Sierra & Dieguez‐Uribeondo 2014) investigated the aetiology of chronic egg mortality events occurring in farmed brown trout, *Salmo trutta,* and identified the causative agent as *Saprolegnia australis*. Yet another study (Cao *et al*. 2012) isolated *Saprolegnia ferax* from infected fish eggs, showing the diversity of species infecting eggs of different fish species.

Appressorial infection structures have been reported before in some *Saprolegnia* species (Willoughby & Hasenjäger 1987). However, we did not observe any appressoria‐like structures by SEM in this study.

In summary, we found that *S. parasitica* and *S. diclina* employ different mechanisms of infection of salmon eggs. For the former, hyphae are able to penetrate an apparently intact chorion, while *S. diclina* is capable of complete destruction of the chorion of salmon eggs. Further studies are needed to elucidate the mechanisms involved.
